# Silicon: A Review of Its Potential Role in the Prevention and Treatment of Postmenopausal Osteoporosis

**DOI:** 10.1155/2013/316783

**Published:** 2013-05-15

**Authors:** Charles T. Price, Kenneth J. Koval, Joshua R. Langford

**Affiliations:** Orlando Health Department of Orthopedic Surgery, 1222 Orange Avenue, Orlando, FL 32806, USA

## Abstract

Physicians are aware of the benefits of calcium and vitamin D supplementation. However, additional nutritional components may also be important for bone health. There is a growing body of the scientific literature which recognizes that silicon plays an essential role in bone formation and maintenance. Silicon improves bone matrix quality and facilitates bone mineralization. Increased intake of bioavailable silicon has been associated with increased bone mineral density. Silicon supplementation in animals and humans has been shown to increase bone mineral density and improve bone strength. Dietary sources of bioavailable silicon include whole grains, cereals, beer, and some vegetables such as green beans. Silicon in the form of silica, or silicon dioxide (SiO_2_), is a common food additive but has limited intestinal absorption. More attention to this important mineral by the academic community may lead to improved nutrition, dietary supplements, and better understanding of the role of silicon in the management of postmenopausal osteoporosis.

## 1. Introduction

Nutrition, exercise, and lifestyle are recognized as important factors in the management of osteoporosis [1, 2]. Dietary supplementation with calcium and vitamin D decreases the risk of fractures and improves the effectiveness of pharmacological management [1, 3–5]. In addition to calcium and vitamin D, a wide range of nutritional supplements have been recommended to improve low bone density, but the evidence of benefit is limited [6, 7]. The absence of evidence may mean that more study is required, or it may mean that supplementation is unnecessary. 

Many essential nutrients behave synergistically, for example, vitamin D and vitamin K in the production and activation of osteocalcin. Vitamin D stimulates the production of osteocalcin, while vitamin K carboxylates osteocalcin for improved bone toughness [8, 9]. Thus, there may be several micro-nutrients that should be supplemented in addition to calcium and vitamin D as part of the management of osteoporosis. The US National Institutes of Health have documented that more than half of the adults in the USA are insufficient in dietary intake of magnesium, vitamin K, vitamin C, and other nutrients that are essential for bone health [10–12]. One mineral that warrants attention is silicon because of the growing body of the scientific literature that recognizes silicon's importance for bone health [13, 14].

Silicon is an essential mineral for bone formation [15, 16]. In 1970, Edith M. Carlisle, Ph.D., published a brief paper in *Science* titled “*Silicon: a possible factor in bone calcification*” [17]. She performed quantitative electron probe analysis of silicon content in young mice and rats. Carlisle concluded that silicon is important as an initiator of mineralization because silicon is highly concentrated in immature osteoid but declines as calcium content rises in mature bone. Another study by Carlisle reported that silicon supplementation accelerates the rate of bone mineralization [18]. She continued her research with several additional studies including a publication in 1981 titled “*Silicon: a requirement in bone formation independent of vitamin D*_1_” [15]. In this study, bones of silicon-deficient chicks contained less collagen than the bones of silicon-supplemented chicks regardless of vitamin D levels. Carlisle concluded that silicon had an effect on collagen to make the bone matrix more calcifiable. The essential nature of silicon for skeletal development was also confirmed by Schwarz and Milne in 1972 and by Nielsen and Sandstead in 1974 [16, 19]. No other researchers reported the role of silicon in bone health until 1993, when Hott et.al. published a study of the effects of silicon supplementation on bone density in ovariectomized rats [20]. They reported that silicon reduced bone resorption and increased bone formation in the animal model of postmenopausal osteoporosis.

Since 2002, there has been increased research regarding the role of silicon in a variety of tissues including bone [13, 14, 21, 22]. The purpose of this report is to review the role of silicon as an essential element for bone formation and maintenance. A secondary purpose is to call attention to this nutritional component so that more research may be directed towards the study of silicon for the management of osteoporosis. It is possible that silicon supplementation should be considered in addition to supplementation with other vitamins and minerals for the management of patients with low bone density.

## 2. Silicon Chemistry

Silicon is a *chemical element*, which has the symbol **Si** and an atomic weight of 28. It is classified as a semiconductor with electrical properties that are intermediate between metal and nonmetal elements. Crystalline silicon has piezoresistive properties that are utilized in micropressure transducers and computer electronics. Silicon rarely occurs as a pure free element in nature. It forms strong bonds with oxygen and generally exists as silica or silicate compounds. Silica is the general term for inorganic compounds containing silicon and oxygen. The silicon dioxide (SiO_2_) form is a major component of sand, granite, quartz, and other types of rocks, clays, and gems in the Earth's crust [22]. Thus, silicon is the second most abundant element in the Earth's crust. Silicon dioxide is poorly soluble in water and has many industrial applications including abrasives, electronics, and construction. Industrial food preparation uses silica powder to decrease foaming, reduce caking of powders, or clarify liquids. Another compound form of silicon is Silicone. Silicones are polymeric compounds with a silicon-oxygen-silicon (Si-O-Si) backbone. These polymers can be linked together to form rubber-like materials that are used for many purposes including plumbing, dental applications, medical implants, tubing, lubrication, and insulation. Neither silicon dioxide nor silicone rubber compounds are useful dietary sources because they have poor water solubility and poor biological availability [22, 23]. In contrast, water-soluble forms of silicon are more biologically available. Silicon in geological formations, especially in volcanic areas, may gradually dissolve to produce soluble forms of silicon in artesian waters [22, 24, 25]. Monomethylsilanetriol (MMST), or CH_3_-Si-(OH)_3_, is a commercially available liquid form of silicon that has biological availability and is used as a liquid nutritional supplement [26].

Water-soluble forms of silicon are absorbed in the intestinal tract, with excess amounts eliminated by the kidneys within 4–8 hours following ingestion [14]. Thus, it is unlikely for silicon to accumulate in excessive amounts in healthy individuals. Oral toxicity from elemental or organic silicon has not been identified in animals or humans even when rats and mice have been fed up to 1000 times the normal dietary intake [26]. Patients on dialysis may accumulate silicon because renal failure prevents the excretion of silicon. Serum silicon levels up to ten times normal have been reported in patients with renal failure, but no adverse effects have been associated with these levels [27, 28]. There have been rare cases of silica renal stones in patients who were also consuming large quantities of magnesium in the form of magnesium trisilicate antacids [22, 29]. Thus, adverse effects from oral silicon have not been observed in healthy individuals. 

## 3. Silicon's Role in Bone Formation

Silicon is bound to glycosaminoglycans and has an important role in the formation of cross-links between collagen and proteoglycans [15, 30, 31]. Silicon is present in all body tissues, but the tissues with the highest concentrations of silicon are bone and other connective tissue including skin, hair, arteries, and nails [14]. In vitro studies have demonstrated that silicon stimulates type 1 collagen synthesis and osteoblast differentiation [32]. Studies in rats have demonstrated that silicon at physiological levels improves calcium incorporation in bone when compared to rats that are deficient in silicon [20, 33, 34]. Thus, silicon is an essential element for bone formation [15, 16].

The exact sequence of mineralization is unknown, but Carlisle concluded that silicon probably acts by making the bone matrix more calcifiable [15]. Silicon concentrations in osteoid are 25 times greater than in surrounding areas and the silicon content gradually declines as calcification occurs [17]. Silicon is a known semiconductor of electrical charges. Silicon crystals are used in microscopic pressure transducers because they have a piezoresistive effect when subjected to stress [35]. It is also known that the collagen matrix of immature bone has piezoelectric properties that generate electrical potentials when subjected to strain. Bone mineralization occurs in the electronegative areas that are generated by compression [36]. It is possible that silicon plays a role in the electrochemical process of mineralization, but the precise biological role of silicon remains unknown. 

Studies of dietary silicon supplementation in growing animals have reported improved bone quality by direct measurements of bone strength and density for quail, broiler chickens, and rainbow trout [37–39]. A randomized blind study of racing quarter horses compared a control group to three different levels of dietary silicon supplementation [40]. The supplemented horses began their diets at six months of age and continued for 18 months including a six-month training and racing period. Race times, lameness, fractures, and serum silicon levels were recorded during the period of study. At the completion of the study, it was determined that the horses with medium and high levels of silicon supplementation had significantly faster race times and greater training distances before the first breakdown. The horses with the highest level of silicon supplementation also had increased bone mineral density in the third metacarpal [40].

Direct measurements of bone mass and strength in numerous animal models have demonstrated the beneficial effects of silicon supplementation to increase bone mineral density and to reduce bone fragility [20, 33, 37–43]. The ovariectomized rat is a standard model for postmenopausal bone loss [44]. Five reports have been published using this model to study the effects of dietary silicon on bone metabolism [20, 41–43, 45]. Hott et al. compared physiological levels to low levels of dietary silicon in ovariectomized rats [20]. The mineral apposition and bone formation rate was 30% greater in the group with physiological silicon intake. The silicon-supplemented group also had less bone resorption. An experimental study by Calomme et al. demonstrated increased femoral bone density when physiological levels of silicon supplementation were added to the standard diet [45]. Additional research in postmenopausal animal models used high levels of supplemental dietary silicon (20 mg/kg/day) [41–43]. These high levels of silicon stimulated bone formation, increased bone mineral density, and decreased calcium excretion in the urine. This effect of increased bone formation has even been noted in calcium-deficient rat models although calcium supplementation combined with silicon supplementation produced greater bone mineral density [43].

Although silicon supplementation is associated with increased bone mineral density, the exact mechanism for this action has not been identified. Serum measurements of bone turnover have been inconsistent, while markers of bone matrix formation are consistently increased. This may indicate that silicon improves mineralization without affecting the rate of bone formation or bone loss. There may also be an effect on collagen that improves bone strength independent of mineral density [46]. In contrast to studies reporting improved bone strength, two experimental studies in rats have reported small reductions in bone strength when excessively high and prolonged levels of dietary silicon were added to the diet [43, 47]. This may represent an antagonistic effect of excessive silicon that decreases intestinal absorption of calcium and magnesium when very high amounts of silicon are provided in the diet [47]. 

Silicon also has biological activity for bone formation when incorporated into calcium phosphate bioceramics [48–50]. These bioceramic materials are used as bone graft substitutes to augment or replace autogenous bone grafts for orthopedic surgical procedures. Calcium phosphate ceramics without silicon substitution are considered osteoconductive because they provide a scaffold for resorption and replacement by bone through osteoclastic resorption and osteoblastic deposition of new bone [36]. Substitution of less than 1% of the phosphate groups (PO_4_) with silicate ions (SiO_4_) enhances the biological activity of the material [48, 50] and creates osteoinductive properties. Coathup et al. compared implantation of calcium phosphate to implantation of silicate-substituted calcium phosphate into the paraspinal muscles of sheep [48]. The silicate-substituted calcium phosphate demonstrated osteoinductive properties and significantly increased the amount of bone that formed compared to the calcium phosphate implants. The osteoinductive properties of silicate-substituted calcium phosphate ceramics have been reported by other researchers [50]. The exact mechanism of this enhanced bone formation is uncertain [49, 51]. One explanation is that silicon in the ceramic generates a more electronegative surface that promotes bone formation. Another explanation is that elemental silicon is released during resorption of the ceramic material and directly stimulates the differentiation and proliferation of osteoblasts. Regardless of the mechanism of action, there is an increasing confirmation that silicon plays a role in bone formation. 

## 4. Osteoporosis and Silicon Intake

Average daily dietary intake of silicon is 20–50 mg for European and North American populations [14]. Daily intake of silicon is higher in China and India (140–200 mg/day) where grains, fruits, and vegetables form a larger part of the diet [52, 53]. China and India also have the lowest prevalence of hip fractures compared to all other regions of the world [54].

Diets containing more than 40 mg/day of silicon have been positively associated with increased femoral bone mineral density compared to dietary intake of less than 14 mg/day [55]. A study of postmenopausal Scottish women determined that average daily intake of silicon was 18.6 mg/day which was lower than a standard British diet that contains approximately 30 mg/day [56, 57]. Dietary intake of silicon declines with age by approximately 0.1 mg/year [58]. In a North American epidemiological study, none of the postmenopausal women achieved 40 mg/day of dietary silicon intake [55].

Two epidemiological studies have reported the relationship between dietary silicon intake and osteoporosis [55, 59]. Increased silicon intake correlated with increased bone mineral density for men, premenopausal women, and postmenopausal women on hormone replacement therapy (HRT). Silicon intake and bone mineral density did not correlate for post-menopausal women who were not on HRT [55]. Macdonald et al. noted that estrogen status may be important for silicon metabolism and suggested that silicon and estrogen may interact synergistically [21]. However, this does not explain the increased bone mineral density in men with increased silicon intake, and the amount of dietary silicon intake by postmenopausal women was generally low. None of the postmenopausal groups achieved more than 40 mg/day of dietary silicon intake which is the amount associated with increased bone mineral density in men and premenopausal women. It is known that estrogen increases the intestinal absorption of calcium [60] so, it is possible that estrogen also influences the intestinal absorption of silicon. Thus, there may be a role for silicon supplementation to increase silicon absorption in post-menopausal women who are not on HRT, but more research is needed to determine the link between estrogen and silicon. 

Silicon supplementation has had limited study as a method to increase bone mineral density in women with postmenopausal osteoporosis [61, 62]. Intramuscular injections of silicon as monomethyl trisilanol at a dose of 50 mg twice a week for four months were administered to postmenopausal women with osteoporosis. This treatment was compared to etidronate, fluoride, magnesium, and controls [61]. Patients in all groups received 1000 mg of calcium and 500 IU of Vitamin D daily. A significant improvement in femoral bone density was noted in the silicon group compared to the other groups. Vertebral bone density improved more with administration of magnesium and etidronate than with silicon. This is consistent with a study of silicon supplementation in ovariectomized rats that demonstrated a significant increase in femoral bone mineral density and only marginal increases in lumbar bone density [45].

Another study of silicon supplementation in women with osteoporosis evaluated change in trabecular bone volume measured by iliac crest biopsy following a period of treatment [62]. Three groups consisted of controls, parenteral administration of 16.5 mg/wk of silicon for four months, or oral supplementation with 27.5 mg/wk for three months. Participants consumed their normal diets, but supplemental calcium or vitamin D was not added. The two groups with supplemental silicon had significant increases in trabecular bone volume compared to the control group [62]. 

A more recent study was conducted in osteopenic women using 3, 6, or 12 mg of silicon supplementation compared to controls [46]. All four groups received calcium and vitamin D supplementation but no other forms of treatment. After one year, the control group had a decrease in femoral bone density, while the groups with silicon supplementation maintained bone density. However, the difference was not statistically significant. It should be noted that average dietary silicon intake ranges from 20–50 mg per day in the United States; so, the levels of silicon supplementation in this study was low, even for the 12 mg/day group. 

Serum markers of bone turnover instead of direct measurements of bone mineral density have also been studied following silicon supplementation [46, 63]. These studies have been inconclusive. One was a short-term study of 12 weeks that did not show any measurable changes [63]. The other study reported a significant positive change in the markers for type I collagen formation (PINP) but no change in other markers of bone turnover [46].

Based on these reports in postmenopausal women and in experimental models of postmenopausal osteoporosis, there is evidence that moderate silicon supplementation has a beneficial effect on bone mineralization and bone density, that is, independent of other factors. 

## 5. Dietary Sources of Silicon

Principle sources of dietary silicon are whole grains, fruits, beverages, and vegetables in that order [14, 22, 56, 64] (Table 1). Unrefined cereals and grains have high silicon content, especially oats and oat bran. Rice hulls and husks are rich sources of silicon. Beer has high silicon content due to the processing of barley and hops. Meats, dairy products, and refined flours have little silicon content. Drinking water can be a source of silicon depending on the source and method of processing [14, 24]. Hard water typically has higher silicon levels than soft water. Initial purification of drinking water by flocculation decreases silicon content in tap water [22, 64].

The relationship between bone density and consumption of beer, wine, and liquor was evaluated by Tucker et al. [65]. They found that moderate consumption of alcohol was associated with increased bone mineral density in men and postmenopausal women when the source of alcohol was beer or wine but not when the source was liquor. This suggests that components other than alcohol may influence bone density. Another study demonstrated that nonalcoholic beer acutely reduced markers of bone resorption [66]. However, the same study demonstrated that moderate intake of ethanol alone also decreased markers of bone resorption. Although Tucker and Sripanyakorn et al. suggested that the silicon in beer had a moderate effect on bone formation independent of ethanol, the short-term effects of silicon ingestion on markers of bone resorption could not be demonstrated [65, 66].

The bioavailability of silicon for intestinal absorption depends on the solubility of the silicon compound [22, 58]. Silicon levels are high in bananas but the silicon is highly polymerized and poorly absorbed [24]. Absorption of silicon is best from whole grains and grain products (breakfast cereals, breads, rice, and pasta). The silicon uptake from green beans and dried fruits is intermediate [58]. Orthosilicic acid is soluble and absorbable form of silica, that is, present in beer, some beverages, and some drinking water. High levels of orthosilicic acid are found in natural sources of water from volcanic areas [58].

Silicon is also available in some nutritional supplements with varying amounts of bioavailability [24]. Poor absorption is noted for antacids containing silicon such as magnesium trisilicate. Supplemental monomethyl silanetriol (MMST) is an absorbable form of silicon while choline-stabilized orthosilicic acid is intermediate. In general, the smaller molecules, or monomeric forms, are better absorbed than the larger, highly polymerized, or oligomeric forms [24].

In the absence of evidence of oral toxicity in animals or humans, safe upper levels for humans have been recommended with a maximum range of  700–1,750 mg/day [22, 26]. Thus, it is unlikely that modest nutritional supplementation would cause adverse effects in humans with normal renal function.

## 6. Summary

Optimum therapy for postmenopausal osteoporosis includes a balanced approach of prevention, exercise, nutrition, early diagnosis, and appropriate treatment. While there are numerous factors that contribute to bone health and to therapy for postmenopausal osteoporosis, silicon is also a mineral that is increasingly recognized as an essential nutrient for bone formation and maintenance. More attention to this important nutrient by the medical community may lead to improved dietary supplements and better understanding of the role of silicon in management of postmenopausal osteoporosis.

## Figures and Tables

**Table 1:** Common dietary sources of silicon [58, 64].

Dietary source	Portion size	mg/portion
Beer	12 oz	8.25 mg
Red wine	4 oz	1.70 mg
Raisins	100 gm	8.25 mg
Green beans	250 gm	6.10 mg
High-bran cereal	100 gm	10.17 mg
Whole grain bread	200 gm	4.50 mg
Mineral water	0.5 L	0–40 mg depending on brand
Brown rice with husks	100 gm	2.07 mg
